# Effect of Massoia (*Massoia aromatica* Becc.) Bark on the Phagocytic Activity of Wistar Rat Macrophages

**DOI:** 10.3390/scipharm86020019

**Published:** 2018-05-10

**Authors:** Triana Hertiani, Agustinus Yuswanto, Sylvia Utami Tunjung Pratiwi, Harlyanti Muthma’innah Mashar

**Affiliations:** 1 Department of Pharmaceutical Biology, Faculty of Pharmacy, Universitas Gadjah Mada, Sekip Utara, Yogyakarta 55281, Indonesia; sylvia_pratiwi@ugm.ac.id; 2 Centre for Natural Anti-Infective Research, Faculty of Pharmacy Universitas Gadjah Mada, Sekip Utara, Yogyakarta 55281, Indonesia; 3 Department of Pharmaceutical Chemistry, Faculty of Pharmacy, Universitas Gadjah Mada, Sekip Utara, Yogyakarta 55281, Indonesia; ag_yuswanto@ugm.ac.id; 4 Politeknik Kesehatan Kemenkes Palangka Raya, Jl. George Obos No. 30/32, Palangka Raya 73111, Indonesia; harlyanti@poltekkes-palangkaraya.ac.id

**Keywords:** immunomodulatory, *Massoia aromatica* Becc., macrophage phagocytosis

## Abstract

The essential oil of Massoia (*Massoia aromatica* Becc., Lauraceae) bark is a potential immunomodulator in vitro. This study evaluated the potential immunomodulatory effects of Massoia bark infusion on the nonspecific immune response (phagocytosis) of Wistar rats. For the in vitro assay, macrophages were treated with the freeze-dried infusion at the concentrations of 2.5, 5, 10, 20, or 40 µg/mL media. For the in vivo assay, two-month-old male Wistar rats were divided into five groups. The baseline group received distilled water at the dose of 1 mL/100 g body weight (BW), with the herbal product containing *Phyllanthus niruri* extract that was administered as the positive control at the dose of 0.54 mL/rat. The treatment groups received the infusion at a dose of 100, 300, or 500 mg/100 g BW. Treatments were given orally every day for 14 days. The ability of macrophage cells to phagocyte latex was determined as phagocytic index (PI), and it was observed under microscopy with 300 macrophages. The in vitro study revealed that the phagocytic activity of the infusion-treated macrophages significantly increased in comparison with that of the control macrophages in a concentration-dependent manner. Among all of the treatment concentrations, the concentration of 40 µg/mL provided the highest activity with a PI value of 70.51 ± 1.11%. The results of the in vivo assay confirmed those of the in vitro assay. The results of the present study indicate that Massoia bark can increase the phagocytic activity of rat macrophage cells.

## 1. Introduction

Immunomodulatory agents affect mechanisms that are related to the pathophysiology and etiology of various diseases by regulating the immune system. An immunomodulator is required to activate the host defense mechanism in response to immune disruption [1,2,3] or to boost immune response against bacterial pathogens [1]. 

Massoia (*Massoia aromatica* Becc., family Lauraceae) is a plant that originates from Papua and Maluku. Local people use parts of this plant, particularly its boiled or steamed bark and trunk, as a traditional treatment for fever and inflammation, and to deter fungi and insects [4,5,6]. The aqueous infusion that is derived from boiled Massoia bark may contain C-10 massoilactone as an essential component. Sa’roni and Adjirni (1999) reported that the infusion exerts a mild anti-inflammatory effect in mice when it is administered at a dose of 300 mg/100 g body weight (BW) [6]. The analgesic activity of Massoia bark infusion when being administered at the dose of 100 mg/10 g BW is comparable with that of acetosal that is administered at the dose of 0.52 mg/10 g BW [7].

The bark contains essential oil with C-10-massoialactone as the major constituent [6,8]. Our previous in vitro experiment revealed that Massoia essential oil and C-10 massoialactone enhance the phagocytic activity of mouse macrophages [9]. However, no study has evaluated the effect of Massoia bark infusion as an immunomodulator on the nonspecific immune response (phagocytosis) of Wistar rats. The infusion or water extract has been chosen to prepare the sample in order to imitate the traditional usage of this Massoia bark by the local people. 

## 2. Materials and Methods

### 2.1. Equipment

Oven (Memmert, Schwabach, Germany), freeze dryer (Benchtop Pro, Warminster, CA, USA), Thin Layer Chromatography scanner (Camag, Muttenz, Switzerland), laminar air flow hood (Labconco, Fort Scott, KS, USA), micropipette (Socorex, Ecublens, Switzerland), vortex (Shimadzhu, Kyoto, Japan), centrifuge (Sorvall, Mountain view, CA, USA), hemocytometer (Neubauer, Karlsruhe, Germany), 24-well microplates (Nunc, Saint Neots, England), 5% CO_2_ incubator (Heraeus, Hanau, Germany), light microscope (Olympus, Hamburg, Germany), inverted microscope (Olympus, Hamburg, Germany), and cover slips (SPL, Gyeonggi-do, Korea).

### 2.2. Materials

*M. aromatica* bark was collected from Sorong, Papua. Taxonomy identification was performed at the Laboratory of Pharmacognosy, Dept. of Pharmaceutical Biology, Faculty of Pharmacy, Universitas Gadjah Mada (UGM) and registered as Nr. BF/3507/Ident/I/2016. Pre-coated Thin Layer Chromatography (TLC) silica gel F254 plates (Merck, Darmstadt, Germany), ethyl acetate, toluene (Pro Analyses, Merck, Darmstadt, Germany), Roswell Park Memorial Institute (RPMI) medium (Sigma-Aldrich, Hamburg, Germany), fetal bovine serum (FBS) (Gibco, New York, NY, USA), Fungizone (Gibco, New York, NY, USA), penicillin–streptomycin (Pen-Strep) (Sigma-Aldrich, Hamburg, Germany), latex (3 μm) (Sigma-Aldrich, Hamburg, Germany), Phosphate Buffer Saline (PBS) (Gibco, New York, NY, USA), and Giemsa (Merck, Darmstadt, Germany). A commercial herbal preparation syrup containing *Phyllantus niruri* extract (25 mg/5 mL) was used as positive control for in vivo study in this research. 

### 2.3. Animal Testing

Male Wistar rats with an age of two months old were bred in the Integrated Research and Testing Laboratory (LPPT), Universitas Gadjah Mada. The handling of laboratory animals was approved by the Commission of Ethical Clearance for Preclinical Research LPPT UGM with Nr. 315/KEC-LPPT/VIII/2015.

### 2.4. Sample Preparation

**Figure 1:**
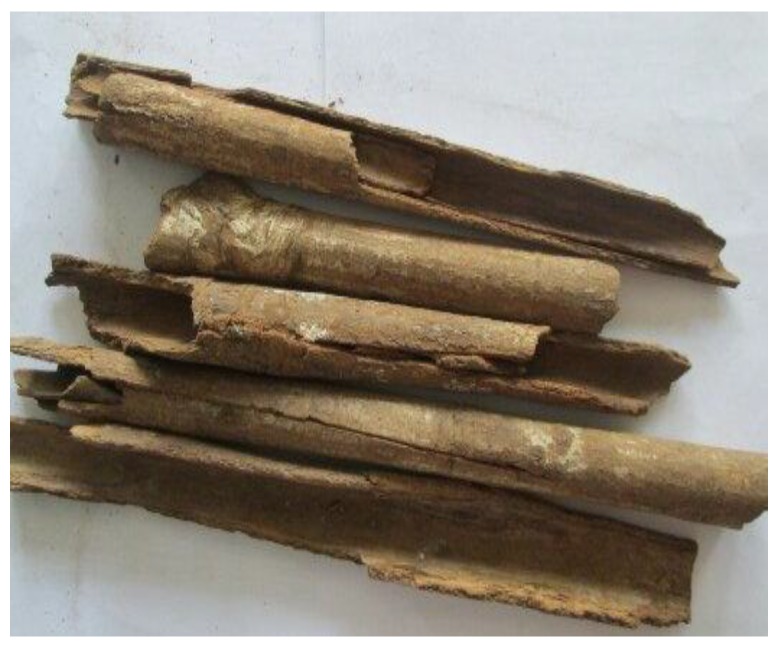
*Massoia aromatica* Becc. bark.

Bark samples (Figure 1) were dried in an oven at 40 °C–60 °C, powdered, and infused in accordance with the Indonesian Pharmacopoeia [6,7]. In vitro tests were performed by using dried extract (WEM) that was prepared from freeze-dried 10% infusion (10 g pulverized barks in 100 mL distilled water). Dried extract was used to make the serial dilution in RPMI medium for sample preparation in the in vitro assay. In vivo testing was performed using 20% infusion prepared fresh daily to imitate the application as traditional herbal tea 20 g pulverized barks in 100 mL distilled water. The infusion for the in vivo assay was prepared in a higher dose in order to reduce the volume of sample that was applied to the tested animal. 

### 2.5. Phytochemical Analysis

The WEM was screened for phenolics, flavonoid, and alkaloid contents by using TLC covering FeCl_3_, AlCl_3_, and Dragendorf as spraying reagent, respectively, for detection. Tube analyses were performed to analyze the presence of saponin (saponification) and tannin (gelatin test). In order to determine the C-10 massoialactone content, WEM was fractionated with ethyl acetate–water (1:1 *v*/*v*) to obtain the ethyl acetate fraction (FEM). Following solvent evaporation, the FEM TLC profile was analyzed by using a pre-coated silica gel F254 plate as the stationary phase and toluene: ethyl acetate (93:7 *v*/*v*) as the mobile phase. Quantitative analysis was performed with TLC scanner at 211 nm by using C-10 massoialactone isolate as a standard. TLC analysis densitometry was performed using TLC silica gel F254 plates that were measuring 20 cm × 10 cm. A total of 3 mL of sample was spotted on a TLC plate with a distance of 1 cm between spots, and was then eluted in a saturated twin-trough chamber for a distance of 8 cm. 

### 2.6. Macrophage Phagocytosis Assay

Animals that were used for the in vitro assay were directly sacrificed without pretreatment. For the in vivo test, animals were divided into five groups. Each group consisted of five rats and received different treatments, namely: (1) 1 mL/100 g BW baseline distilled water; (2) 0.54 mL/rat positive control; (3) 100 mg Massoia bark infusion/100 g BW; (4) 300 mg Massoia bark infusion/100 g BW; and (5) 500 mg Massoia bark infusion/100 g BW. The infusion of 20% Massoia bark was given orally once a day for 14 days. Dose regimens were prepared, as follows: 100 mg/100 g BW for a 150 g rat refers to 0.75 mL infusion (20% *w*/*v*); 2.25 mL for 300 mg/100 g BW; and, 3.75 mL for 500 mg/100 g BW. The animals were sacrificed on the 14th day of treatment for analyses. 

#### 2.6.1. Macrophage Isolation

Macrophages were isolated by injecting approximately 10 mL cold RPMI 1640 into the peritoneal cavity of each rat. Aspirates were centrifuged at 1200 rpm for 10 min. Then, 3 mL of complete RPMI media (containing 10% FBS) were added to each pellet. Cells were counted with a hemocytometer and suspended in complete media to obtain a cell suspension with a density of 2.5 × 10^4^ cells. The cell suspension was placed in a 24-well micro titer plate that was covered with round cover slips (5 × 10^3^ cells/wells). The cells were incubated in a 5% CO_2_ incubator at 37 °C for 30 min. Complete medium was added to each well. The plates were then incubated for 24 h [10].

#### 2.6.2. Latex Test for Phagocytic Activity

Phagocytic activity was tested using latex discs 3 µm in diameter and was suspended in PBS to reach 2.5 × 10^6^ particles/mL. For the in vitro testing, macrophage culture, as described in 3.3.1, after incubated for 24 h was washed twice with RPMI. The WEM in RPMI was added at to reach concentration of 2.5, 5, 10, 20, or 40 µg/mL to each wells. The macrophage cultures were incubated again for 4 h in a 5% CO_2_ incubator. The cells were washed thrice with PBS. Afterwards, 500 µL latex suspension was added. The plates were then incubated in a 5% CO_2_ incubator at 37 °C for 1 h. The cells were washed thrice with PBS to remove excess latex. Cells were fixed with methanol for 10 min. The cover slips were allowed to dry and were then stained with 20% *v*/*v* Giemsa for 20 min. The number of macrophages that engulfed latex, as well as the amount of latex that was engulfed by macrophages was counted under microscopy to calculate the phagocytic index (PI) [11]. For the in vivo test, the latex suspension was added directly after the macrophage culture had been incubated for 24 h and was washed twice with PBS. The rest of the assay steps were similar to those of in vitro testing. The PI was calculated using this formula below [11]:

(1) $$\mathrm{PI}=\left(\frac{\mathrm{amount}\;\mathrm{of}\;\mathrm{latex}\;\mathrm{engulfed}}{\mathrm{amount}\;\mathrm{of}\;\mathrm{total}\;\mathrm{macrophage}}\right)\times \left(\frac{\mathrm{amount}\;\mathrm{of}\;\mathrm{macrophage}\;\mathrm{engulfed}\;\mathrm{latex}}{\mathrm{amount}\;\mathrm{of}\;\mathrm{total}\;\mathrm{macrophage}}\right)\times 100$$

### 2.7. Data Analyses

Data were analyzed for homogeneity and normality using SPSS 16.0 to determine if parametric or nonparametric test statistical analysis should be conducted. Normally and homogeneously distributed (*p* > 0.05) data were analyzed through one-way analysis of variance. Data that were not normally or homogeneously distributed (*p* < 0.05) were analyzed through the Kruskal Wallis test, followed by Mann–Whitney test. *p* < 0.05 was considered significant.

## 3. Results

Phytochemical screening of the WEM detected only the essential oil components and tannin, whereas flavonoids and alkaloids were not detected by TLC using AlCl_3_ and Dragendorf spraying reagents. Tube test for saponin showed a negative result, while those on tannin showed a positive result. The C-10 massoialactone content of the ethyl acetate fraction of the Massoia infusion was measured through TLC densitometry. The C-10 massoialactone content of 10% and 20% FEM was 0.29 and 0.70 mg/mL (8.13% and 12.21%), respectively. 

**Figure 2:**
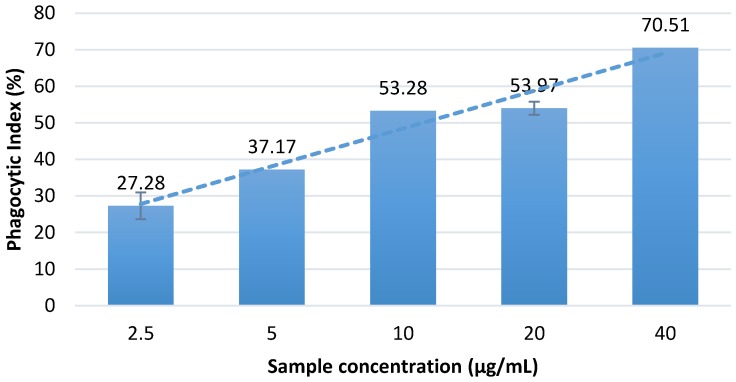
Phagocytic index of macrophages following treatment with dried extract (WEM) in vitro (n = 3; *α* = 0.05). Normal control showed phagoytic index (PI) 5.60 ± 1.36. Results showed that treatment with the Massoia bark infusion significantly increased macrophage PI as compared with treatment with the control. The highest increase in PI was observed under treatment with 40 µg/mL Massoia bark infusion (PI = 70.51 ± 1.11). PI increased in a dose-dependent manner.

The in vitro assay showed that the aqueous Massoia bark extract can significantly increase the phagocytic activity of macrophages in comparison with the normal control having PI value of 5.60 ± 1.36 (Figure 2). Among all of the treatment dosages, treatment with 40 µg/mL Massoia bark infusion showed the highest ability to enhance the phagocytic activity of macrophages. Concentration-dependent activity was observed. 

**Figure 3:**
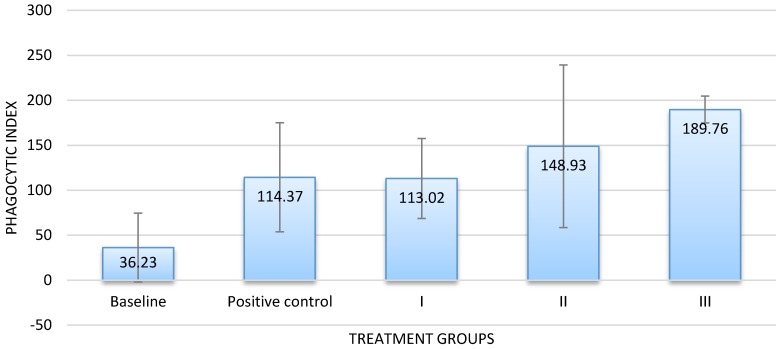
Phagocytic index of macrophages against latex after treatment with Massoia bark infusion in vivo (n = 5, *α* = 0.05). The in vivo phagocytic activity of macrophages under treatment with 20% infusion administered at the dose of 100 (I), 300 (II), or 500 mg/100 g BW (III) was studied. All of the test groups showed a tendency of increase in the macrophage phagocytosis activity in comparison to the baseline. However, only groups under treatment with 300 mg and 500 mg /100 g BW, phagocytic activity increased to levels that were significantly different from those of the baseline and those under treatment with the positive control.

The results of the in vivo assay corresponded with those of the in vitro assay. Groups that received the infusion at dose of 300 or 500 mg/100 g BW exhibited a higher PI value than the baseline and the positive control groups. By contrast, the PI value of groups that received the infusion at a dose of 100 mg/100 g BW was almost similar to that of the positive control (Figure 3). Dose-dependent activity was also confirmed.

## 4. Discussion

The results of the in vitro assay supported the results of Hertiani et al. [9], who reported that an increase in the phagocytic activity of macrophages is related to the increase in the content of C-10 massoialactone. The infusion method can be used to efficiently extract the active compound from the bark of the Massoia plant. As a comparison, the essential oil was reported to contain 46.00% of C-10 massoialactone [12], while the infusion (20%) can contain 0.70 mg/mL (12.21%) by boiling 20 g bark in 100 mL water. 

It is noteworthy that the infusion at 100 mg/ 100 g BW and at 300 mg/100 g BW tested showed no significant difference with the commercial product, while the sample at 500 mg/100 g BW caused a significantly higher stimulatory effect on the macrophage phagocytosis. *Phyllanthus niruri* which is the active ingredient of the commercial product used as the positive control has been widely used as herbal remedy and has been reported to be an effective immune stimulant [13]. By conversing the doses of Massoia infusion used on rats in this research, we calculated that the doses that were given to human would be 9.73, 29.19, and 48.65 g/60 kg BW. That refers to application of 20% Massoia bark infusion in volumes of 48.65, 145.95, and 243.25 mL, respectively. Those support the traditional application by the local people.

Several sesquiterpene lactones [14] have been reported to increase the phagocytic activity of macrophages. C-10 massoialactone, which is the active constituent of the Massoia bark, has an active site that is mainly correlated to its β-unsaturated lactone moiety [15]. However, this compound is relatively toxic to the Vero cell line and human fibroblast primary cells [12]. Its toxicity may thus limit its application. Several β-unsaturated lactones have been reported to exhibit the inhibition of IL-2 production [16]. This may further influence the macrophage activity [17]. Barros et al. also recommend that several (–)-massoialactone derivatives have potential as anti-inflammatory agents by reducing the macrophage production of nitric oxide following lipopolysaccharide (LPS) stimulation [15]. 

Besides containing the C-10 massoialactone, other compounds, such as cinnamaldehyde, cinnamyl acetate, cinnamic acid, eugenol, resin, and tannin were also previously reported from the Massoia bark [6,8]. Our experiment detected the presence of tannin in the Massoia infusion. Tannin has been reported to show immunomodulation activity, for example, an ellagitannin and Oenothein B [18]. However, cinnamaldehyde has been reported to show otherwise [19]. Therefore, it is also interesting to find out whether the tannin and cinnamaldehyde also contribute in modulation of the macrophage activity following sample application. Nevertheless, C-10 massoialactone as the active compound exhibits a broad-spectrum antimicrobial activity against planktonic and biofilm cultures [9]. This activity may enhance the potential value of Massoia bark as an immunomodulator in responses to microbial infection.

## 5. Conclusions

In vitro and in vivo assay results showed that the infusion of Massoia bark can effectively increase macrophage phagocytic activity in comparison to the untreated control. Treatment with the 20% infusion that was administered at the dose of 500 mg/g BW caused significantly higher phagocytosis activity in comparison with those that were treated with the positive control.
